# Medical education and COVID-19: a personal view

**DOI:** 10.3325/cmj.2020.61.213

**Published:** 2020-06

**Authors:** David Oliver

**Affiliations:** 1 Tizard Centre, University of Kent, Canterbury, UK; 2 Visiting Professor, Zagreb University School of Medicine, Zagreb, Croatia *drdjoliver@gmail.com*

The COVID-19 pandemic challenges us all – personally, academically, and within society. The way we interact day to day has been altered, certainly in the short term over the coming months, and maybe in the longer term over the next years. This has affected all areas of life, but education has been particularly affected, as in the past courses have usually been delivered face to face with close interaction between teacher and student.

The greatest change that is developing is the increasing use of online resources. These have already been developing for many years, with the recording of lectures becoming a routine in many universities, so that students could revisit them and use the resources as part of their overall study. However, online lectures have become the main form of teaching and may continue to be needed over the coming months. This does bring some benefits but there are also risks. Care needs to be taken to ensure that true education is provided, with stimulation and encouragement of the students and true learning.

The benefits of online resources include the following:

• The lecture can be optimized so that it is of the best quality possible – teachers can use the opportunity to record the lecture at their convenience and when all the necessary resources are present.

• The possibility of the lecture being watched at a time convenient to the student, and on a repeated basis if this is necessary for full understanding.

• Lectures can be given in real time – with the possibility of interaction, although this is more difficult at a distance.

• New teachers can be involved, even from other areas or countries. At a recent conference in February, just before lockdown, one speaker, from another country, was unable to attend and presented online, with no problems and full interaction, including answering questions.

• Students are often used to online resources and cope very well with the system, minimizing the technical issues.

• Time is used efficiently as there is no travel to and from venues. The future of large international congresses is in doubt as everyone realizes that if the meeting is online, travel and accommodation costs will be eliminated and time away from the workplace reduced. A recent virtual congress of the European Academy of Neurology had over 42 000 participants from all over the World.

• Patients could be included within sessions. They can be recorded, giving their history and views on care as well as having signs demonstrated. This recording can be paced and at their convenience, so that the risks of them becoming overtired is reduced. In this way patients are more likely to agree to being involved. Their input can enhance teaching and be very helpful in engaging students in the session.

• Students could be involved with some areas that they may have difficulty seeing normally – recordings of patients who are usually unable to come to clinics or lectures, recordings of operations, continual recordings over time of a patient showing the development of a disease process, and rural or isolated areas can be involved.

• Online sessions can be shared with other areas – even with other countries, where the availability of educational resources may be more limited (1).

There are also risks and challenges of developing online systems:

• The personal contact and interaction are more complex and difficult to organize. Discussion and cross-fertilization of students, which may happen in an interactive session or seminar, is not as easy. We may all have to consider how this can be facilitated.

• The teaching of practical skills may be more difficult, although there is increasing evidence that basic skills, including communication skills, can be developed virtually (2).

• There will be a need for the development of skills for both teachers and students in the use of online resources. It may not be as simple as recording “the usual lecture,” but a new approach may need to be developed. These skills will be necessary for teachers, but students may also need to develop skills in the best use of virtual teaching.

• Lectures may need to be carefully planned and extra techniques used, such as video clips, to ensure that students do not become bored and lose attention.

• Students may become isolated. Student-to-student interactions are important, and need to be facilitated.

• It is essential that resources are kept up to date – the same lecture cannot necessarily be used every time, with new student groups.

There are areas that may be particularly challenging. Within the teaching of palliative care, the aim is often to help students develop communication skills, consider and develop their attitudes and feelings in care, and to look at how they do interact with patients, families, and other members of the multidisciplinary team. Teaching these issues may not be insurmountable, but care is needed to ensure students are able to discuss some issues in greater depth, and a blended approach, of online teaching with small face-to-face seminar groups, may be necessary.

These are great challenges, but with openness to change, and a desire to move forward, online resources may become firmly developed with the curriculum. However, we must never lose the opportunity for face-to-face discussion, interaction and sharing of experiences, as these are crucial in the development of a student into a doctor.

## References

[1] Memon AR, Rathore FA (2018). Moodle and online learning in Pakistani Medical Universities: an opportunity worth exploring in higher education and research.. J Pak Med Assoc.

[2] Kyaw BM, Posadzki P, Paddock S, Car J, Campbell J, Tudor Car L (2019). Effectiveness of digital education on communication skills among medical students: systematic review and meta-analysis by the Digital Health Education Collaboration. J Med Internet Research.

